# Medicinal Plants for the Treatment of Hypertrophic Scars

**DOI:** 10.1155/2015/101340

**Published:** 2015-03-11

**Authors:** Qi Ye, Su-Juan Wang, Jian-Yu Chen, Khalid Rahman, Hai-Liang Xin, Hong Zhang

**Affiliations:** ^1^College of Life Science, Fujian Agriculture and Forestry University, Fuzhou 350002, China; ^2^Department of Pharmaceutical Botany, School of Pharmacy, Second Military Medical University, Shanghai 200433, China; ^3^Central Laboratory, Shanghai Seventh People's Hospital, Shanghai 200137, China; ^4^State Key Laboratory of Quality Research in Chinese Medicine, Macau Institute for Applied Research in Medicine and Health, Macau University of Science and Technology, Macau; ^5^School of Pharmacy and Biomolecular Sciences, Faculty of Science, Liverpool John Moores University, Liverpool L3 3AF, UK; ^6^Department of Pharmacognosy, School of Pharmacy, Second Military Medical University, Shanghai 200433, China

## Abstract

Hypertrophic scar is a complication of wound healing and has a high recurrence rate which can lead to significant abnormity in aesthetics and functions. To date, no ideal treatment method has been established. Meanwhile, the underlying mechanism of hypertrophic scarring has not been clearly defined. Although a large amount of scientific research has been reported on the use of medicinal plants as a natural source of treatment for hypertrophic scarring, it is currently scattered across a wide range of publications. Therefore, a systematic summary and knowledge for future prospects are necessary to facilitate further medicinal plant research for their potential use as antihypertrophic scar agents. A bibliographic investigation was accomplished by focusing on medicinal plants which have been scientifically tested *in vitro* and/or *in vivo* and proved as potential agents for the treatment of hypertrophic scars. Although the chemical components and mechanisms of action of medicinal plants with antihypertrophic scarring potential have been investigated, many others remain unknown. More investigations and clinical trials are necessary to make use of these medical plants reasonably and phytotherapy is a promising therapeutic approach against hypertrophic scars.

## 1. Introduction

Scar formation strongly depends on the presence of contraction during healing and the nature of the scar is actually the uneven look of the healed tissue resulting from disfigured tissue deformation and overaligned collagen fibers [1]. Collagen in hypertrophic scars is found to be in a disorganized, whorl-like arrangement rather than in the normal parallel orientation manner. Therefore, hypertrophic scars are indurate, elevated, poorly extensible, and also characterized by hypervascularity, thereby providing their erythematous appearances [2]. HS can cause significant abnormality in aesthetic and functional symptoms and to date no recognized treatment has been established. It commonly occurs after surgical incision, thermal injury, and traumatic injuries to the dermis with a subsequent abnormal healing response [3]. Furthermore, it is often associated with contractures that can lead to considerably reduced functional performance in patients.

The development of antihypertrophic scars is an unsolved problem in the process of scar treatment. For this reason, some undiscovered successful treatments are needed to prevent excessive hypertrophic scarring. The reported preventions include topical medical application, cryotherapy, use of silicone gel sheets, injection of steroids, radiotherapy, and an early surgical procedure for wound closure [2]. In the last decade, there has been a renewed interest in the use of indigenous medicine worldwide, arising from the realization that orthodox medicine is not widespread. Although modern medicine may be available in some communities, herbal medicines have often maintained popularity for historical and cultural reasons, in addition to their cheaper costs [4]. Recent research has introduced the uses of phytochemical compounds and extracts isolated from medicinal plants in an attempt to resolve these problems as a promising therapy.

Many treatment strategies are sought to prevent scar formation without compromising the wound healing process [5]. The effectiveness of currently used therapy against hypertrophic scar arises most probably from the increase of the medicinal plants reported. In the modern system of medicine, about 25% of prescriptions contain active principle(s) derived from plants [4]. A significant correlation between medicinal plants and their use in the treatment of many types of scars has been shown in epidemiological data generated throughout the world. Published clinical trials have, as yet, largely focused on characterizing the pharmacokinetics and metabolism of medicinal plants. Despite experimental advances in medicinal plant research against scars, findings in humans are still limited. However, in recent years, diverse benefits of medicinal plants in the treatment of hypertrophic scars have been described [6–9].

In line with the latest findings responsible for the increased recognition of medicinal plants as potential therapeutic and/or preventative agents, the aim of the present review is to focus on recent experimental findings and clinical trials of medicinal plants and other preparations with similar actions that could account for beneficial effects on hypertrophic scars in patients. Natural products, such as plant extracts, either as pure compounds or as standardized extracts, provide unlimited opportunities for control of hypertrophic scarring owing to their chemical diversity [10]. Currently, a great deal of effort is being expended to find alternative sources of safe, effective, and acceptable natural medicinal plants for the treatment or prevention of hypertrophic scars; hence, all literature available was reviewed.

## 2. Suggested Mechanism of Hypertrophic Scarring

**Figure 1:**
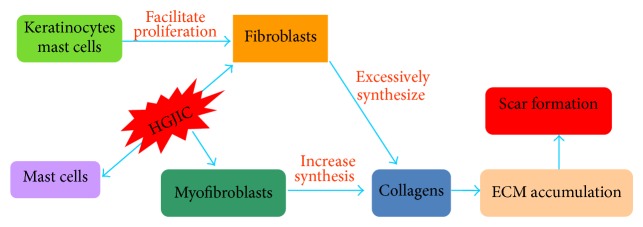
The mechanism of hypertrophic scarring.

The molecular mechanism of hypertrophic scarring is associated with the unusual proliferation of fibroblasts and overproduction of collagen and extracellular matrix [70]. An array of intra- and extracellular mechanisms is essential in the prevention of scar formation. With the help of molecular biology, cell biology technology, hypertrophic scar animal models, and the setting-up of scar tissue engineering, the mechanism of hypertrophic scarring has been clearly defined (Figure 1). It is usually considered as migration and proliferation of different cell types such as keratinocytes, myofibroblasts [59], and mast cells [71]. Fibroblasts play an essential role in new tissue formation during wound repair [33], but their abnormal low death rate and high proliferation rate can cause scar tissue formulation [11]. Meanwhile, keratinocytes are indispensable in signal transduction between paracrine secretion and epithelium matrix. When cultured in the presence of keratinocytes, fibroblasts exhibit significant proliferation activity [72], showing the contribution of keratinocytes to fibroblasts proliferation. Myofibroblasts, which are different from fibroblasts and are related to the composition, organization, and mechanical properties of ECM [73], increase collagen synthesis and retard cell migration [71], thus resulting in excessive and rigid scarring. Fibroblasts are transformed into myofibroblasts by heterocellular gap junction intercellular communications between mast cells (RMC-1) and fibroblasts [71, 74]. In the process of wound healing, the combination of fibroblasts and myofibroblasts triggers excessive production of abnormal extracellular matrix protein [75], eliciting scarring [1, 75]. With the assistance of keratinocytes and mast cells, proliferative fibroblasts produce massive collagen which makes extracellular matrix accumulate below dermis, leading to scar formation. The complex forming process consists of three different phases, inflammation, proliferation, and maturation, which leads to hypertrophic scarring in the end [76]. The ratio of I to III collagens in healthy adults ranges from 3.5 to 6.1, while in patients with hypertrophic scars, it could be down to 2 and in keloid patients it can be as high as 19, which is related to the abnormal metabolism of collagens I and III in pathological scars, including more collagen synthesis and less collagen degradation.

**Figure 2:**
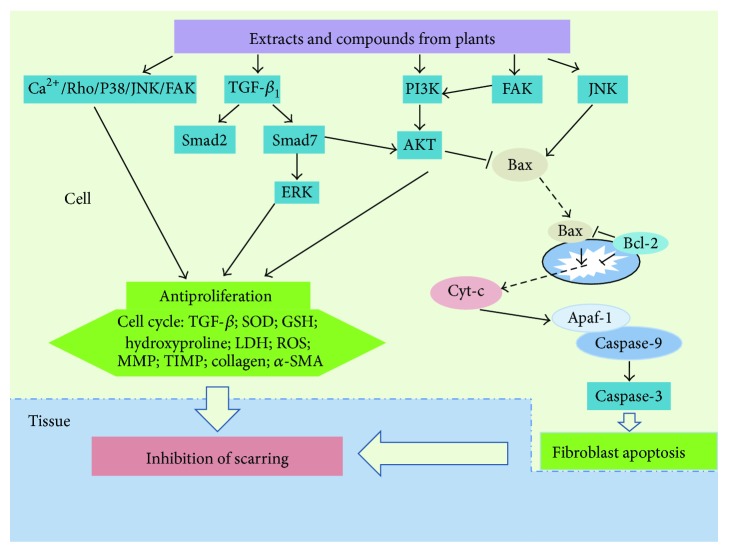
The mechanisms by which extracts and compounds from medicinal plants display antihypertrophic scar activity.

Although many targets of action, by which scarring can be inhibited, have been experimentally studied or postulated, few are well known or defined for inhibition of hypertrophic scarring by plant-derived compounds.  Figure 2 and Tables 5 and 6 summarize and enumerate the suggested mechanisms and correlative medicinal plants.

The size of a scar is influenced by many factors, such as wound size, wound contraction, and healing time. Wound contraction makes an important contribution to scar formation also the larger the area of the wound, the more cells migrate, resulting in more prominent scarring [1]. Therefore, induction of fibroblast apoptosis and reduction of extracellular matrix and collagen I/III production may be the pivotal measures against hypertrophic scarring.

Many kinds of test models are applied to investigate wound healing mechanisms and inhibition of scar formation, including 2D hybrid agent-based model [1], pig surgical injury model, fibroblast populated collagen lattice (FPCL) model, rat laminectomies at Lumbar-1 level [5], incisional wound healing model [6], and rabbit ear model [54]. These models provide a mean for detecting and evaluating the mechanobiology in wound healing and scar formation [1].

However, the complex mechanism of hypertrophic scarring still remains unknown which raises the question of how to control scar hyperplasia.

## 3. Medicinal Plants against Hypertrophic Scarring

Many beneficial uses of medicinal plants are extensively documented in the traditional medicine systems in many cultures. To collect the data which supports this finding, we performed a systematic review using PubMed, Elsevier, Springer, and Google Scholar databases and peer-reviewed articles published in the last 10 years. The search terms included scar, scaring, fibroblast, extract, and preparation. The phytochemicals from medicinal plants against scar hyperplasia are presented in Tables 1 and 2, respectively, whilst the medicinal plant extracts are listed in Table 3. Their activities and mechanisms for antihypertrophic scarring were also described, respectively, in Tables 1, 2, and 3. There are five preparations (Table 4) reported on their effects and mechanisms of antihypertrophic scarring, namely, liposome-encapsulated 10-HCPT, oxymatrine-phospholipid complex (OMT-PLC), solid lipid nanoparticle-enriched hydrogel (SLN-gel), Ginsenoside Rg3/poly (l-lactide) (G-Rg3/PLLA), and* Centella asiatica* extract capsule, which are composed of different medicinal plants and vehicles. Medicinal plants can be used for different therapeutic purposes or as precursors of useful drugs containing different types of phytochemicals.

The use of herbal medicine remedy has been steadily increasing worldwide in recent years, as well as the search for new phytochemicals that could be potentially developed as useful drugs for the treatment of hypertrophic scar and other scar diseases [4]. The antihypertrophic scar activity of medicinal plants results from a variety of components contained in these plants (Tables 1 and 2). Many plant extracts (Table 3) have antihypertrophic scar activity owing to their phytochemical constituents. However, more work is needed to focus on purification and identification of active components and to elucidate the roles that these play in inhibition of scars when used alone or jointly. Moreover, many of them have not been tested for their cytotoxicity to normal cells, which seriously blocks* in vivo* investigations. Undeniably, no toxic and side effects have been proved for some active components. For example, Genistein, which is easily obtained and commonly used for hypertrophic scar treatment, has strong pharmacological effects, with no obvious toxicity or side effects [13].

## 4. New Preparations of Medicinal Plants

A large number of extracts and compounds of medicinal plants display antiscar activity. Nevertheless, drugs are difficult to get through the stratum corneum due to the natural barrier of skin, which causes lower permeability of drugs. The oral bioavailability of drugs at the permissive dose is very low, owing to their hydrophilicity (low permeability), poor absorption, and biotransformation or compact scar tissue. The appropriate form of prepared drugs can evidently improve drug permeability, lipid solubility, skin permeability, retention ratio, release time, and cytotoxicity. Hydroxycamptothecin (HCPT) is thought to be one of the most effective components against scars. However, the poor solubility and short half-life severely limit its clinical applications [65]. Compared with HCPT, the liposome-encapsulated HCPT (L-HCPT) can reduce epidural fibrosis by preventing the proliferation of fibroblasts in the scar tissue with longer half-life and better solubility [65]. The application of a silicone derivative to herbal extracts can improve skin pliability and alleviate the concomitant symptoms of scars including pain and itching [2]. However, it is extremely important to control the cytotoxicity of biomaterials for their clinical applications. Microemulsion, a transparent dispersion system, is a good vehicle for drug delivery due to its many advantages such as thermodynamic stability (long shelf life), easy formation (zero interfacial tension), low viscosity, high surface area (high solubilization capacity), and small droplet size [67]. It has been revealed that drug-free microemulsion is a promising preparation due to inapparent cytotoxicity [67]. The local or transdermal application of water-soluble pharmaceutical formulation may be suitable for medicinal plant extracts and compounds.

Owing to compact scar tissue, it is necessary for the combination of natural products or crude extracts with some adjuvant as new dosage forms to increase their solubility, content, release time, uptake, and penetrability. These dosage forms include microemulsion [67], liposomes [66], solid lipid nanoparticle [68], and electrospun fibrous scaffolds [29]. Improvement of drug permeation may be a promising treatment in future research on the basis of the known medicinal plants.

In addition, some of these plant extracts or purified chemical components are prepared as traditional medicinal injections for the deep antiscar treatment. For example,* Carthamus tinctorius* injection, whose primary component is hydroxysaflor yellow A, softens hypertrophic scar tissue and inhibits fibroblast proliferation by decreasing the type I/type III collagens ratio and the TGF-*β*_1_ level after local treatment [77]. The* radix astragali* injection also inhibits the proliferation and reduces scar thickness and hardness by reducing Smad3 and TGF-*β*_1_ levels [78].

## 5. Current Treatment and Prospects for Future Therapies

Currently, occlusive dressings, compression therapy, intralesional steroid, cryosurgery, laser, radiation, surgical excision, and interferon therapy are curative for the majority of patients with hypertrophic scars [79]. Surgical therapy and excising fiber fraction are the common approaches for the treatment of hypertrophic scars. However, significant disadvantages were reported, such as the recurrence of adhesion after surgery as high as 45%–100% [54], which seriously limits its extensive application to scar prevention. Accordingly, physiotherapy is established, including occlusive dressings, pressure therapy, cryosurgery, radiation therapy, and laser therapy. Meanwhile, pharmacotherapy is also frequently applied, such as intralesional corticosteroid injection and topical drug treatment with interferon, bleomycin, 5-fluorouracil, verapamil, vitamin E, imiquimod, TGF-*β*_3_, or interleukin-10 [79, 80]. Pharmacotherapy mainly inhibits inflammation, proliferation, and remodeling phase [7] or modifies ECM metabolism via interfering the pivotal molecules of MAPK, TGF-*β*, and PI3K signaling transduction.

However, there is no ideal treatment for hypertrophic scars so far and some chemical drugs also cause mal-effects simultaneously. Many kinds of natural products from medicinal plants have good antiscar activity and show notable advantages due to their fewer side-effects. Therefore, in addition to widespread uses of surgical therapy, physiotherapy, and pharmacotherapy, there is a great need for developing new natural drugs more efficient than or synergizing with the existing ones. Many kinds of purified natural products originated from medicinal plants are abundant in the natural environment, such as Ginsenoside Rg3 [29], Oleanolic Acid [54], Resveratrol [42], Asiaticoside [34], and Genistein [13], and are popular as antiscar agents due to their easy obtainment and fewer side-effects. Hence, we overviewed the major current herbs and their preparations applied to the treatment of hypertrophic scars.

It is a challenge to identify and evaluate a safe, wholesome, and effective natural product against scars. Even though a number of new products have been reported by pharmacological tests in the last decades, many others remain unknown or untested.

## 6. Discussion

In this review, we gathered publications on medicinal plants with antihypertrophic scar activity and addressed the question whether the treatment of scars with medicinal plants is effective in humans. Although* in vivo* and* in vitro* investigations play an important role in the evaluation of safety and effectiveness of medicinal plants in preclinical trials, there is no perfect denouncement for their ultimate success as human drugs. Clearly, animal data are not sufficient for the confirmation of the safety and efficacy of medicinal plants in humans owing to their physiological structure differences. Furthermore, there are some conflicting clinical trials reported. For example, it has been reported that honey was effective in rapidly cleaning infection and promoting wound healing, indicating that honey possessed anti-infection activity [81]. However, it was also reported that honey did not affect the wound, scar, length, and remained length [82]. Therefore, the effectiveness of some drugs needs to be further clarified.

On the other hand, only four publications reported negative results in our retried papers. Genistein phosphorylated c-Raf, MEK1/2, ERK1/2, and p38 proteins, but not JNK protein [14]. Asiaticoside had no effect on the expression of Smad2, Smad3, and Smad4 [34], while madecassoside regulated keloid-derived fibroblasts proliferation, migration, F-actin filaments, cytoskeletal protein actin, and the phosphorylation of cofilin via p38 MAPK and PI3K/AKT signaling, but not ERK1/2 and caspase-8 signaling [12]. Quercetin promoted phosphorylation of JNK and ERK, but not p38; it increased the protein and mRNA expression of MMP-1, but not type I collagen and TIMP-1 [38]. These studies indicate that the antiscar activity of medicinal plants needs to be scrutinized further.

Many traditional medicines used in folk medicine are reported to have antiscar activity, but only a few have been studied systematically* in vitro* or/and* in vivo*, such as rhubarb [60] and tamarind [63]. Although numerous* in vitro *studies have substantiated the antiscar activity of plant extracts and phytochemicals, there is very little evidence in humans. The number of clinical trials and their highlighted results are limited. The numerous traditional formulations effectively and extensively used in clinics have not been investigated. Also, the majority of the plants (Tables 1, 2, and 3) traditionally used as antiscar agents have not been investigated in animals. The phytochemicals with* in vitro* antiscar activity may have no effects* in vivo* due to the exceedingly high doses. Moreover, many of these phytochemicals have not been tested for their cytotoxicity, acute toxicity, or/and long-term toxicity in normal cells and animals, which seriously limits* in vivo* investigations. Only two medicinal plants have been reported on their untoward reactions and cytotoxic effects. The clinical efficacy and safety should be investigated simultaneously for medicinal plant extracts and compounds.

The natural barrier of skin can block drug getting through stratum corneum or decrease the amount of drug permeation, causing inefficiency or low-efficiency of drugs. Some adjuvants can significantly improve the penetrability of drugs and the desired therapeutic effects can be achieved. For example, hydroxycamptothecin (HCPT) is considered one of the most effective agents against scars, which prevents fibroblast proliferation and reduces epidural adhesion, but the poor solubility and short half-life severely limits its clinical application [65]. Some new dosage forms evidently reverse these conditions, such as microemulsion [67], liposomes [66], solid lipid nanoparticle [68], and electrospun fibrous scaffolds [29]. Therefore, the development of new dose types is necessary in order to ameliorate drug effects.

Although enormous progress has been achieved over the last years, the impact of medicinal plants on individual types of scars needs to be explored in more detail. Polymechanistic phytochemicals such as Genistein may have an advantage over targeted therapeutics, which simultaneously tackle scar treatment from multiple angles. Genistein can act on many target points, including suppression of PDGF-promoted TPK activation, decrease of types I/III precollagen and PCNA expression, reduction of c-Raf, MEK1/2, ERK1/2, and p38 protein phosphorylation, and inhibition of RTK-Ras-MAPK (ERK/p38) [13]. Further insights into the molecular mechanisms of phytochemicals will facilitate the development of new drugs for the prevention and treatment of human scars.

## 7. Conclusion

In conclusion, the scaring process is complicated. The characteristics of an appropriate therapy for the prevention and treatment of scars should comprise the following: simple and easy delivery, comparability (effectiveness) with current therapies, and minimal drug interaction with concomitant treatments and lack of significant side effects [83]. Manyextracts and compounds from medicinal plants can inhibit scarring. The main mechanisms are suppression of proliferation and/or induction of apoptosis in scar fibroblasts by regulation of several pathways, such as MAPK, PI3K/AKT, RhoA/ROCK-I, VEGF, FAK, and TGF-*β*/Smad. Although the approaches described here are quite different and mechanisms are complicated, the utility should be maximized for medicinal plants as antihypertrophic scar agents. However, screening is necessary to minimize any potentially harmful side effects on human skin and health.

## Figures and Tables

**Table 1:** The components from medicinal plants with antihypertrophic scar activity.

Component	Botanical name	Family	Medicinal part	Observation	Dose	Effect	Mechanism of action	References
Madecassoside	*Centella asiatica *	Umbelliferae	Whole plant	*In vitro *	10~100 *μ*M	Antiproliferation of HSFBs Promotion of apoptosis Diminishment of scar formation Facilitation of wound healing	Inhibition of HKF migration, F-actin filaments protein, and cytoskeletal protein. Promotion of nuclear shrinkage and mitochondrial membrane depolarization Condensation of chromatin and fragment of nuclei Inhibitory phosphorylation of p38, PI3K, AKT, and cofilin. Activation of caspase-3/caspase-9 Facilitation of Bax mRNA expression and decrease of Bcl-2 and MMP-13 mRNA expression	[11, 12]

Genistein	*Glycine max *	Leguminosae	Fruit	*In vitro *	25~100 *μ*g/mL	Anti-proliferation of HSFBs Suppression of mitosis Promotion of apoptosis	Inhibition of TPKs, increase of caspase-3, and decreases of *α*-SMA and Bcl-2 protein Enhancement of Bax protein Inhibition of types I/III precollagen mRNA expression, down-regulation of collagen I/III mRNA, reduction of PCNA expression, and inhibitory phosphorylation of c-Raf, MEK1/2, ERK1/2, and p38 Induction of morphology changes of apoptosis cells Inhibitory transdifferentiation of fibroblasts into myofibroblasts	[13–17]
					37~370 *µ*M		Decrease of G_0_-G_1_ phase and increase of G_2_-M phase Increase of C-JUN mRNA expression and decrease of FOS-B mRNA expression in skin keratinocytes Inhibitory mRNA expression of C-JUN and C-FOS in human fibroblasts In keloid fibroblasts, decrease of C-JUN and C-FOS mRNA expression at 37 *µ*M, but enhancement at 370 *µ*M	

Astragaloside IV	*Astragalus Membranaceus *	Leguminosae	Root	*In vitro *	12.5~200 *μ*M	Antiproliferation of HSFBs	Decrease of collagen I/collagen III and TGF-*β*_1_ secretion	[18]

Tetrandrine	*Stephania tetrandra *	Menispermaceae	Root	*In vitro*	10~80 *μ*M	Antiproliferation of HSFBs	Inhibition of TGF-*β*_1_ mRNA transcription, promotion of Smad7 and MMP-1 mRNA expression, and inhibition of Smad2 mRNA expression Decrease of protein expression of collagen I/collagen III, Bcl-2, and MKP-1. Reduction of total collagen volume and S phase, increase of G_0_/G_1_ phase, and prevention of G_0_/G_1_ into G_2_ phase Inhibitory phosphorylation of MEK1/2 and ERK1/2	[19–22]
				*In vivo*	1~10 mg/L			
				Local injection	0.5~2 mg/L 50 mg/mL, 20 *μ*L			

Aloe-emodin	*Rheum palmatum *	*Polygonaceae *	Root, rhizome	*In vitro *	20~80 mg/L	Antiproliferation of HSFBs	Increase of S phase	[23]

5F	*Pteris semipinnata *	Pteridaceae	Whole plant	*In vitro*	20~80 *μ*g/mL	Antiproliferation of HPS	Blockage of fibroblasts from G_1_ to S phase Decreased protein expression of TGF-*β*_1_ and type I collagen, increase of caspase-3, and reduction of total collagen and fibroblasts PCNA protein (cyclin) Inhibitory mRNA expression of type I/type III procollagen in SSSF	[24–26]
				*In vivo*	10~40 mg/L	Reduction of PS volume		
					40~120 mg/L	Antiproliferation of SSSF		
				Local injection	20~80 mg/L	Promotion of HPS apoptosis Decrease of hypertrophic index	Reduction of collagen fiber content	

Oxymatrine	*Sophora japonica *	Leguminosae	Root	*In vitro *	0.125~1.0 mg/mL 2 *μ*M	Antiproliferation of KFb and HFb Promotion of KFb apoptosis	Increase of S phase, inhibitory mRNA expression of collagen I/collagen III and reduction of protein expression of Smad3 and ERK_1_ Promotion of Smad7 protein expression Inhibition of p-Smad3 and nuclear translocation of Smad3	[27, 28]

Ginsenoside Rg3 (G-Rg3)	*Panax ginseng *	Araliaceae	Root, rhizome	*In vivo* Local injection	3 mg/mL, 0.1 mL	Inhibition of HS Decrease of scar tissue fibrosis	Increase of protein expression of PCNA, Bax, caspase-3, and Cyt-c Decrease of Bcl-2 protein expression	[29, 30]

Osthole	*Cnidium monnieri *	Apiaceae	Fruit	*In vitro *	5~50 *μ*M	Antiproliferation of HSFBs and Induction of apoptosis	Promotion of Bax mRNA expression and inhibition of Bcl-2 mRNA expression Decreases of TGF-*β*_1_ protein expression and facilitation of HSFBs shrinkage, chromatin condensation, membrane blebbing, apoptotic body formation, and DNA ladder formation	[31]

**Table 2:** Antihypertrophic scar displaying phytochemicals widely distributed in medicinal plants.

Phytochemicals	Observation	Dose	Effect	Mechanism of action	References
10-Hydroxycamptothecin (HCPT)	*In vivo *	0.01~0.1 mg/mL	Decrease of the area of epidural scar tissue and the number of fibroblasts. Reduction of epidural adhesion and inhibitory proliferation of RESF	Inhibition of topoisomerase I	[5]

Angelica naphtha	*In vitro *	1~16 mg/L	Antiproliferation of HSFBs and induction of HSFBs apoptosis	Inhibition of G_0_/G_1_ and G_2_/M phases, promotion of S phase, and reduction of collagen protein in fibroblasts	[32]

Asiaticoside	*In vivo* *In vitro *	25~50 mg/mL Local injection 25~1000 *μ*M 300 *μ*g/mL	Reduction of scar hyperplasia of HSRE Decrease of hypertrophic index Promotion of keratinocytes migration Anti-proliferation of HSFBs	Inhibition of the mRNA expression of TGF-*β*_1_, RhocA, ROCK-I, and CTGF, facilitation of TGF-*β*_3_ mRNA expression, and decrease of the expression of types I/III collagen and TIMP-1 proteins	[33–36]

Matrine	*In vitro *	0.01~5.00 g/L	Antiproliferation and induction of apoptosis in HSFBs	Promotion of G_2_-M phase, inhibition of lactate dehydrogenase and Hyp and enhancement of I/III collagen ratio	[37]

Quercetin	*In vivo*	0.05%~1%, w/o Local Application	Inhibition of scarring in hairless mice	Increase of the protein and mRNA expression of MMP-1 and enhancement of the phosphorylation of JNK and ERK	[38]
	*In vitro *	10~40 *μ*M	Antiproliferation of HSkF		

Emodin	*In vitro *	50~200 *μ*g/mL	Antiproliferation of HSFBs	Inhibition of G_0_/G_1_ phase, increase of intracellular calcium, and decrease of collagen synthesis	[39–41]

Resveratrol	*In vitro* *In vivo *	25~400 *μ*M 150~400 *μ*M Local injections	Antiproliferation of HSFBs Reduction of hypertrophic scar index	Inhibition of the mRNA expression of type I/type III procollagens	[42]

Tan IIA	*In vitro *	20~80 *μ*g/mL 0.05~0.15 mg/mL	Antiproliferation of HSFBs Induction of HSFBs apoptosis	Facilitation of nuclei shrinkage, condensation and fragmentation, blockage of HSFBs from G_1_ to S phases, downregulation of MDA content and XOD activity, increase of T-SOD and GSH-Px activity, and promotion of MMP-1 mRNA expression	[43–45]

Curcumin	*In vitro*	12.5~100 *μ*M	Antiproliferation of HSFBs	Inhibition of procollagen 1 mRNA expression Reduction of hypertrophic index and collagen fiber area density	[46]
	*In vivo *	0.5~2.0 mM, 0.1 mL/d Local injections			

Dihydroartemisinin	*In vivo *	180 mg/kg	Inhibition of HSRE scarring	Inhibition of collagen fibers and hypertrophic index	[47]
		10 mL intragastric administration	Antifibroblast proliferation of HSRE		

Arteannuin	*In vitro*	0.103~0.206 mg/mL	Antiproliferation of HSFBs	Congregation of nuclear chromatin, promotion of calcium concentration, increase of G_0_-G_1_ phase, and reduction of collagen levels and hypertrophic index of HSRE	[48–51]
	*In vivo *	60 mg/mL/2 d	Decrease of HSRE scarring		
		20 *μ*L local injection	Antiproliferation of mastocyte		

Panax notoginseng saponins (PNS)	*In vitro *	400~800 *μ*g/mL	Antiproliferation of HSFBs	Inhibition of G_2_-M and G_0_-G_1_ phases, increase of S phase, reduction of the protein expression of TGF-*β*_1_ and *α*-SMA, and inhibition of intracellular free calcium concentration	[52, 53]

Oleanolic Acid	*In vivo *	Topical application of 2.5, 5, and10% for 28 consecutive days	Inhibition of hypertrophic scarring, induction of apoptosis, and reduction of scar elevation index	Inhibition of the mRNA expression of TGF-*β*_1_ mRNA, MMP-1, TIMP-1, and P311. Increase of the mRNA expression of MMP-2, caspase-3, and caspase-9. Reduction of the protein expression of TGF-*β*_1_ and collagen I/collagen III	[54]

Hirudin	*In vitro *	1~50 *μ*M	Promotion of apoptosis	Increase of G_l_ phase and inhibition of S phase Enhancement of the protein expression of MMP-2, MMP-9, and p27, reduction of the protein expression of cyclin E and TGF-*β*_1_, and inhibition of the mRNA expression of I/III procollagens	[55]

Xiamenmycin	*In vivo *	10 mg/kg·d^−1^, intraperitoneal injection for 10 days	Attenuation of hypertrophic scarring and suppression of local inflammation in a mechanical stretch-induced mouse mode	Reduction of CD^4+^ lymphocyte and monocyte/macrophage retention in fibrotic foci Blockage of fibroblast adhesion with monocytes. Inactivation of FAK, p38, and Rho guanosine triphosphatase signaling	[56]
	*In vitro*	5–30 *μ*g/mL	Inhibition of proliferation of HSFBs		

**Table 3:** The extracts from medicinal plants displaying anti-hypertrophic scarring.

Extract	Botanical name	Family	Medicinal part	Observation	Dose administration	Effect	Mechanism of action	References
Ethanolic extract	*Calotropis gigantea *	Asclepiadaceae	Root, bark	*In vivo *	100~400 mg/kg intragastric administration	Increase of wound contraction and decrease of scar area and the time of epithelization	Increase of hydroxyproline and collagen synthesis	[6]

Ethanolic extract	*Daucus carota *	Apiaceae	Root	*In vivo *	1, 2, and 4% epidermal administration	Decrease of wound area, epithelization period, and scar width. Increase of wound contraction	Increase of hydroxyproline content. Antioxidant and antimicrobial activities	[7]

Methanolic extract	*Pistia stratiotes *	Araceae	Leave	*In vivo *	5 and 10% epidermal administration	Decrease of wound area	Inhibition of hydroxyl radical scavenging and increase of fibroblast blood vessels and collagen fibers	[8]

Ethyl acetate extract	*Gelidium amansii *	Gelidiaceae	Whole plant	*In vitro *	5~10 mg/mL	Antiproliferation of HSFBs	Decrease of the protein expression of I/III collagens and TGF-*β*_1_	[9]

Ethanolic extract	*Carthamus tinctorius *	Asteraceae	Flower	*In vitro *	2~8 *μ*g/mL	Antiproliferation of HSFBs	Inhibition of collagen protein synthesis and promotion of fibroblast shrinkage	[57]

Aqueous extract	*Oenothera paradoxa *	Onagraceae	Seed	*In vitro *	0.1~10 *μ*g/mL	Protection of normal dermal fibroblasts	Decrease of LDH and ROS	[58]

Aqueous extract	Cigarette Smoke	Unknown	Unknown	*In vitro *	100% saturated solution	Antiproliferation of skin fibroblasts and promotion of cellular senescence	Inhibition of SOD and GSH-Px and promotion of ROS	[59]

Ethyl acetate extract	*Rheum palmatum *	Polygonaceae	Root, rhizome	*In vitro *	25 *μ*g/mL	Antiproliferation of HSFBs	Increase of G_0_/G_1_ phase	[60]

Methanol extract	*Broussonetia kazinoki *	Moraceae	Bark, root	*In vitro *	Unknown	Inhibition of hyperpigmentation	Reduction of tyrosinase enzyme synthesis	[61]

Ethanol extract	*Scutellaria baicalensis Georgi *	Lamiaceae	Root	*In vivo *	10 mg/mL epidermal administration	Inhibition of scarring	Reduction of the protein expression of TGF-*β*_1_	[62]

Aqueous extract	*Allium cepa *	Liliaceae	Corm	*In vivo*	1~2.5%, v/v local application	Suppression of scarring in hairless mice	Upregulation of MMP-1 and type I collagen expression	[38]
				*In vitro *	1~2.5%, v/v	Antiproliferation of fibroblasts		

Aqueous extract	*Tamarindus indica *	Fabaceae	Bark, leave	*In vivo *	Unknown	Anti-inflammation	Elimination of death cells and necrotic tissues	[63]

Ethanol extract	*Aneilema keisak *	Commelinaceae	Whole plant	*In vitro *	40 *μ*g/mL	Decrease of scarring	Inhibition of TGF-*β*_1_-dependent signalling by reducing Smad2 protein. Reduction of various hKF pathological responses, including hyperplastic growth, collagen production, and migration without DNA damage	[64]

**Table 4:** The preparations from different medicinal plants with antihypertrophic scar activity.

Preparations	Botanical name	Family	Medicinal part	Preparation	Vehicle	Delivery system	Observation	Effect	Mechanism of action	References
Hydroxycamptothecin (HCPT)	*Camptotheca acuminata *	Nyssaceae	Fruit, leave	Liposome-encapsulated 10-HCPT	Liposome	Liposome-encapsulated	*In vivo* Implant	Antiproliferation of fibroblasts and reduction of epidural adhesion	Decrease of epidural scar area and fibroblast number in the epidural scar tissue	[65, 66]

Oxymatrine (OMT)	*Sophora flavescens*, *Sophora alopecuroides*, and *Sophora subprostrata *	Leguminosae	Unknown	Oxymatrine-phospholipid complex (OMT-PLC)	Phospholipid	Microemulsion	*In vitro* *In vivo* topical delivery	Antiproliferation of fibroblasts	Improvement of OMT skin permeability and increase of retention ratio of OMT in skin.	[67]

Astragaloside IV	*Astragalus* *membranaceus *	Leguminosae	Root	Solid lipid nanoparticle-enriched hydrogel (SLN-gel)	Lipid hydrogel	Solid lipid nanoparticle, hydrogel	*In vitro* *In vivo, *topical delivery	Enhancement of keratinocytes migration and proliferation Increase of drug uptake in fibroblasts Promotion of wound healing and inhibition of scar formation	Caveolae endocytosis pathway. Increase of wound closure rate and angiogenesis Improvement of collagen regular organization	[68]

Ginsenoside Rg3 (G-Rg3)	*Red Panax ginseng *	Araliaceae	Root, rhizome	Ginsenoside Rg3/Poly (l-lactide) (G-Rg3/PLLA)	Electrospun poly (L-lactide) fiber	Electrospun fibrous scaffolds, nanofibers	*In vitro* *In vivo *	Inhibition of fibroblast cell growth, antiproliferation of fibroblasts, and prevention of scar formation	Improvement of dermis layer thickness, collagen fibers, and microvessels	[29]

Centella asiatica extract	*Centella asiatica *	Apiaceae	Whole plant	*Centella asiatica* extract capsule	Capsule	Nothing	*In vivo *	Inhibition of tissue overgrowth, reduction of scar and keloid, and anti-inflammation	Promotion of collagen I protein expression, collagen remodeling, and glycosaminoglycan synthesis Enhancement of collagen and acidic mucopolysaccharides	[69]

**Table 5:** Summary of antiscarring mechanisms of medicinal plant components.

Mechanism		Medicinal plant component
MAPK pathway	Inhibition of p-p38 signaling	Madecassoside, Genistein, and Xiamenmycin
	Inhibition of p-ERK1/2 signaling	Genistein, Tetrandrine, Cryptotanshinone, and Quercetin
	Inhibition of p-JNK signaling	Quercetin
PI3K/AKT signaling		Madecassoside
Mitochondrial-dependent pathway	Increase of Bax	Madecassoside, Genistein, Ginsenoside Rg3, and Osthole
	Decrease of Bcl-2	Madecassoside, Genistein, Tetrandrine, Ginsenoside Rg3, and Osthole
	Increase of cytoplasm Cyt-c	Ginsenoside Rg3
Cell cycle	Decrease of G_0_-G_1_ phase	Genistein, Angelica naphtha, Emodin, and Panax notoginseng saponins
	Increase of G_2_-M	Genistein
	Decrease of S phase	10-Hydroxycamptothecin, Tetrandrine, Aloe emodin, and Hirudin
	Prevention from G_0_/G_1_ into G_2_ phase	Tetrandrine
RhoA/ROCK-I signal pathway	Inhibitory secretion of RhocA, ROCK-I, and CTGF	Xiamenmycin
VEGF signal pathway		Cryptotanshinone
FAK signal pathway		Cryptotanshinone, Xiamenmycin
TGF-*β*/Smad signaling pathway		Oxymatrine
Downregulation of collagen I/III expression		Genistein, Astragaloside IV, Tetrandrine, Resveratrol, 5F, Curcumin, Oleanolic Acid, and Hirudin
Decrease of *α*-SMA		Genistein, Panax notoginseng saponins
Activation of caspases	Activation of caspase-3	Madecassoside, Genistein, 5F, Cryptotanshinone, Oleanolic Acid, and Ginsenoside Rg3
	Activation of caspase-9	Madecassoside, Oleanolic Acid
Suppression of TPK activation		Kazinol F
Inhibition of topoisomerase I		10-Hydroxycamptothecin
Decrease of TGF-*β*_1_ secretion		Tetrandrine, Panax notoginseng saponins, Osthole, and Hirudin
Inhibition of TGF-*β*_1_ transcription		Astragaloside IV, Oleanolic Acid
Downregulation of TIMP-l expression		Oleanolic Acid
Reduction of LDH and increase of the ratio of collagen I/collagen III		Matrine
Increase of T-SOD and GSH-Px activity		Tan IIA
MMP	Enhancement of MMP-1	Tetrandrine, Tan IIA, and Oleanolic Acid
	Enhancement of MMP-2 and MMP-9	Hirudin
	Enhancement of MMP-13	Madecassoside
Increase of intracellular calcium		Emodin, Arteannuin

**Table 6:** Summary of antiscarring mechanisms of plant extracts.

Mechanism	Medicinal plants extract
Cell cycle	
Increase of G_0_-G_1_ phase	Rhubarb
Collagen	
Downregulation of collagen l expression	*Gelidium amansii *
Downregulation of collagen III expression	*Gelidium amansii*, *Scutellaria baicalensis Georgi *
Enhancement of collagen synthesis	*Calotropis gigantea *
Inhibition of collagen synthesis	*Carthamus tinctorius *
Promotion of collagen I	Onion
MMP	
Enhancement of MMP-1	*Neonauclea reticulata*, Onion
Increase of MMP-3 and MMP-9	*Neonauclea reticulata *
Elimination of hydroxyl radical	*Pistia stratiotes *
Decrease of LDH	*Oenothera paradoxa *
Decrease of ROS	*Oenothera paradoxa*, *Neonauclea reticulata *
Increase of ROS and reduction of SOD and GSH-Px	*Cigarette Smoke *

## Abbreviations

AKT: Protein kinase B
ECM: Extracellular matrix
ERK: Extracellular regulated protein kinases
FAK: Focal adhesion kinas
HS: Hypertrophic scar
HKF: Human keloids fibroblast
HF: Human fibroblast
HPS: Human pathological scar
HSkF: Human skin fibroblast
HSRE: Hypertrophic scar model of the rabbit ears
LDH: Lactic dehydrogenase
MAPK: Mitogen-activated protein kinase
MMP: Matrix metalloproteinases
NF-*κ*B: Nuclear factor-kappaB
PCNA: Proliferating cell nuclear antigen
PI3K: Phosphatidylinositol 3-kinase
PS: Pathological scar
RESF: Rats epidural scar fibroblasts
ROS: Reactive oxygen species
SOD: Superoxide dismutase
SSSF: Systemic scleroderma skin fibroblast
TIMP-1: Tissue inhibitor matrix metalloproteinase-1
TGF-*β*_1_: Transforming growth factor-*β*_1_
TPK: Tyrosine protein kinases
TxA2: Thromboxane A2.
